# Tobacco exposure linked to Warthin's tumor being the most common benign parotid neoplasm in Veterans: A retrospective cohort study

**DOI:** 10.1177/00368504251399563

**Published:** 2025-11-18

**Authors:** Louis Abarca, Linda K. Green, Ray Y. Wang, Vlad C. Sandulache, David J. Hernandez

**Affiliations:** 1 Bobby R. Alford Department of Otolaryngology – Head and Neck Surgery, 3989 Baylor College of Medicine, Houston, TX, USA; 2 Pathology Section, Diagnostic and Therapeutic Care Line, 20116 Michael E. DeBakey Veterans Affairs Medical Center, Houston, TX, USA; 3 ENT Section, Operative Care Line, Michael E. DeBakey Veterans Affairs Medical Center, Houston, TX, USA

**Keywords:** Salivary gland, Veterans, parotid gland neoplasms, Warthin tumor, pleomorphic adenoma, tobacco

## Abstract

**Objective:**

Parotid gland neoplasms account for a large proportion of benign salivary gland tumors, with pleomorphic adenomas (PA) being the most common in the civilian population, followed by Warthin's tumor (WT). However, the distinct risk profiles of Veterans significantly influence the incidence and prevalence of salivary gland neoplasms. We investigated the diagnostic and management algorithms for benign parotid gland neoplasms in Veterans.

**Methods:**

Retrospective cohort study. Descriptive statistics were calculated using Microsoft Excel. Categorical variables were compared using chi square tests, and were reported with 95% confidence intervals, with statistical significance set at *P* < .05.

**Results:**

The study included 158 patients who underwent fine-needle aspiration (FNA) only, and 88 who had FNA followed by surgical intervention. Most patients were male (91%) and smokers (73%). WT was the most common overall (45%), followed by PA (16%). Patients with FNA or surgical pathology indicating WT were more likely to have a history of over 10 pack-years of tobacco use compared to those with lymphocytic tissue, cysts, or PA (58/68 [85.3%, 95% CI 76.9–93.7%] vs. 36/70 [51.4%, 95% CI 39.6–63.2%], *P* < .05; 39/46 [84.8%, 95% CI 74.4–95.2%] vs. 16/32 [50.0%, 95% CI 32.8–67.2%], *P* < .05). Concordance between FNA and final pathology was 87.5% (77/88; 95% CI, 80.6–94.4%). Surgical patients experienced low complication rates, with sialocele being the most common at 8% (7/88; 95% CI 2.3–13.6%).

**Conclusion:**

Overall, WT was the most prevalent neoplasm and was strongly associated with heavy tobacco use. The high concordance between FNA and surgical pathology underscores the reliability of FNA as a diagnostic tool. These results support the use of FNA as a reliable tool that can guide patient counseling with greater confidence. Furthermore, the low complication rates observed post-surgery reinforce the safety of surgical management.

## Introduction

Parotid gland neoplasms account for 70% of benign salivary gland tumors, with pleomorphic adenoma (PA) being the most common, followed by Warthin's tumors (WT), or papillary cystadenoma lymphomatosum.^1^ As reported across many multi-center reviews, PA accounts for approximately 60–70% of benign salivary gland neoplasms in the general population, whereas WT comprise roughly 15–25%.^1,2^ However, due to variable risk parameters, the distribution of benign salivary gland tumors can vary among populations.^2–4^ Although there are no well-established risk factors that can lead to development of PA,^5^ WT frequency has been linked to tobacco exposure in multiple patient populations.^6,7^ As a result, the pre-test probability of detecting a PA or a WT can vary among patient populations and ultimately impact both diagnostic accuracy and appropriate management of the tumor.

Fine needle aspiration (FNA) is the definitive clinical tool utilized to distinguish between benign and malignant parotid gland processes with generally high sensitivity and specificity.^8,9^ FNA sensitivity and specificity can vary with the complexity of the neoplasm. For instance, WT's heterogeneous features, such as necrotic areas, mucoid background, inflammation, and atypical squamous cells, highlight the diagnostic challenges and show that FNA diagnosis is not always straightforward.^10–12^

We and others previously established distinct characteristics of U.S. Veterans, inclusive of extensive tobacco, particulate, and oxidative stress exposures, which have been shown to impact the epidemiology, behavior, and management of multiple solid malignancies.^13–16^ Veterans are a known vulnerable population due to disproportionately high rates of substance use, unique military-related exposures, and a higher burden of comorbid medical conditions. These factors contribute to unique clinical groups that warrant dedicated studies to inform diagnostic and management strategies tailored to their specific needs. Here we sought to determine whether distinct exposure patterns in a large Veteran cohort might impact the relative distribution of parotid gland neoplasm, and whether our current diagnostic and management algorithms were appropriately adjusted to an altered pre-test probability.

## Methods

This retrospective cohort study was approved by the Institutional Review Board at the Baylor College of Medicine and the Michael E. DeBakey VAMC (H-40168, approved 10/29/2021). The requirement for written informed consent was waived by the board, and the study was conducted in accordance with the ethical standards of the 1975 Declaration of Helsinki, as revised in 2024. The reporting of this study conforms to the Strengthening the Reporting of Observational Studies in Epidemiology (STROBE) guidelines.^17^ We reviewed the records of Veterans who underwent FNA for a parotid mass between January 2018 and June 2024, identifying all eligible patients consecutively during this period. Inclusion criteria consisted of all patients with an FNA-confirmed benign parotid lesion, yielding a total cohort of 246 cases. Exclusion criteria included those whose FNA results indicated malignancy, normal salivary gland tissue, or represented repeat FNA. Non-diagnostic FNA results were retained in the analysis to maintain completeness; only one case was non-diagnostic and was included in the FNA-only cohort. Deidentified data points were extracted from the electronic medical record (EMR) including demographics, history of tobacco use (pack years, as documented on otolaryngology clinic notes), imaging, cytology and surgical pathology results along with clinical outcomes. For complications and recurrence, data was analyzed through August 1, 2024. Patients with missing data for key variables were excluded from statistical analysis to preserve data integrity. For surgical patients, follow-up was calculated from the date of parotidectomy to the last otolaryngology clinic visit. Complication rates on patients lost to follow-up were classified as having no complications, based on the absence of documented adverse events in the EMR.

### Statistical analysis

Continuous variables were summarized as means with standard deviations in Microsoft Excel, and categorical variables were summarized as total number and percentages. Comparisons of categorical variables between groups were performed using two-sided Chi-square tests with statistical significance defined as *P* < .05. Key proportions of clinical interest, including smoking history, FNA and surgical pathology concordance, PET avidity, surgical complication rates, were reported with 95% confidence intervals (CIs) calculated using the Wilson method. Post hoc power analysis was conducted to assess the probability of detecting observed effect sizes at α = .05.

## Results

A total of 278 Veterans who underwent FNA for a parotid mass were identified. The surgical cohort excluded one patient with normal salivary gland tissue and 14 with malignant pathology, while the FNA-only cohort excluded 16 patients with malignancy and one with normal tissue. As shown in Table 1, the final study cohort included 158 patients who underwent FNA alone and 88 who underwent both FNA and surgical excision after exclusions. Overall, patients were predominantly male (91%) and older than 60 years. Although the surgical group was slightly younger on average, this difference was not clinically meaningful and did not appear to influence management recommendations. Other demographic variables, including sex, race, and smoking history, were similar between groups.

**Table 1.** Demographics of Veteran population.

Sex, *n* (%)	FNA only	FNA + surgical	Total
Male (%)	147 (93%)	78 (89%)	225 (91%)
Female (%)	11 (7%)	10 (11%)	21 (9%)
Age, years ± SD	68.97 ± 11.03	68.97 ± 11.03	68.97 ± 11.03
*Race, n (%)*			
White	92 (58%)	55 (62%)	147 (60%)
Black	62 (39%)	27 (31%)	89 (36%)
Other	4 (3%)	6 (7%)	10 (4%)
*Tobacco use, n (%)*			
History of tobacco use	113 (71%)	67 (76%)	180 (73%)
No history of tobacco use	45 (29%)	21 (24%)	66 (27%)

**Figure 1.**
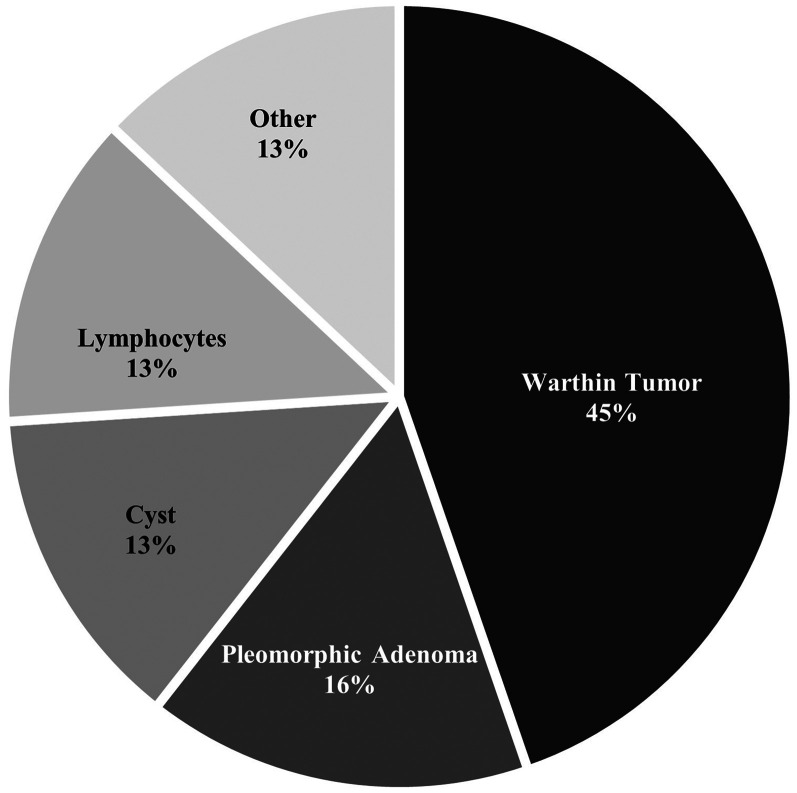
Distribution of benign parotid gland neoplasms in entire cohort.

Tobacco use was common in this patient cohort, with 73% being former or current smokers, and 66% having over 10 pack years. As seen in Figure 1 and Table 2, pathological findings revealed WT as the most common diagnosis in both groups, with 43% (*n* = 68) among FNA patients and 47.7% (*n* = 42) among surgical patients (45% overall). Among FNA only patients, PA had a prevalence of 9.5% (*n* = 15). Among surgical patients, PA was the second most common diagnosis of 27.3% (*n* = 24). PA was the second most common diagnosis at 16% (*n* = 39) among all patients analyzed.

**Table 2.** Distribution of benign parotid neoplasms.

	FNA only patients	FNA—surgical patients	Total
Histology type *N* (%)			
Warthin tumor	68(43.0%)	42(47.7%)	110(44.7%)
Pleomorphic adenoma	15(9.5%)	24(27.3%)	39(15.8%)
Cyst	25(15.8%)	8(9.1%)	33(13.4%)
Lymphocytes	30(18.9%)	2(2.3%)	32(13.0%)
Oncocytic neoplasm	5(3.2%)	4(4.5%)	9(3.6%)
Neoplasm	3(1.9%)	5(5.7%)	8(3.2%)
Inflammation	6(3.8%)	1(1.1%)	7(2.8%)
Abscess	3(1.9%)	0(0%)	3(1.2%)
Chronic sialadenitis	2(1.3%)	0(0%)	2(0.8%)
Lymphoepithelial lesion	0(0%)	1(1.1%)	1(0.4%)
Non-diagnostic	1(0.6%)	0(0%)	1(0.4%)
Lipoma	0(0%)	1(1.1%)	1(0.4%)
Total	158	88	246

As shown in Table S1, WT in the FNA only cohort were significantly more likely to have over 10 pack years of tobacco use compared to those with lymphocytic tissue, cysts, or PA (58/68 [85.3%, 95% CI: 76.9–93.7%] vs. 36/70 [51.4%, 95% CI: 39.6–63.2%]; *P* < .05; power = 0.99). Similarly, in the surgical cohort, 39/46 (84.8%, 95% CI: 74.4–95.2%) of WT cases had >10 pack-years compared to 16/32 (50.0%, 95% CI: 32.8–67.2%) in the other top diagnoses (*P* < .05; power = 0.92). When surgical pathology was available, there was an 87.5% (77/88; 95% CI, 80.6–94.4%) concordance between FNA and final surgical pathology. Imaging showed multifocality in 20% of patients. In the patients who underwent PET scans, incidental avidity was noted in 91% (20/22; 95% CI 72.2–97.5%). Among those with positive avidity, 65% (13/20; 95% CI 43.3–81.9%) were WT. Surgical patients experienced low complication rates, with sialocele/salivary fistula being the most common at 8% (*n* = 7; 95% CI 2.3–13.6%) followed by wound dehiscence at 5% (*n* = 5; 95% CI 0.9–10.5%). There were no instances of recurrence in any of the surgical patients at the time of data collection. Median post-operative follow-up was 2 months, and mean post-operative follow-up was 8.4 months.

## Discussion

Multiple studies report PA frequency exceeding 60% of all tumors, while WT incidence is generally less than 30%. Hussaini et al. analyzed 141 Veterans undergoing FNA, identifying WT as the most common diagnosis (52%) and PA as the second (40%). However, their cohort's tobacco use rate (32%) was significantly lower than ours (73%) and did not look at statistics past distribution of parotid gland neoplasms.^18^ In Veterans, military exposures such as combustion products and occupational chemicals may influence the distribution of parotid gland neoplasms, though no studies have established this correlation. Other environmental factors, including ionizing radiation exposure, obesity and metabolic syndrome associated with chronic inflammation, have been mentioned in the literature but remain less well defined than tobacco exposure and require further validation.^19,20^

In our study, WT was the predominant diagnosis, and unlike other histologies, WT showed a significant association with more than 10 pack-years of tobacco use. FNA proved highly reliable, showing strong concordance with surgical pathology, reflecting cytopathologists’ expertise in recognizing WT's distinct features in this population.

Interestingly, WT's been shown to be FDG-positive, with some hypotheses suggesting that the high concentration of mitochondria, immunoglobulin A, or the accumulation of lymphoid stroma within the tumor may be responsible for this finding.^21^ In our study, some benign, incidental parotid neoplasms exhibited FDG avidity on PET scans performed for unrelated malignancy surveillance, potentially leading to unnecessary malignancy workups. Recognizing FDG avidity in benign tumors allows for more informed discussions with patients regarding prognosis.

Parotidectomy was well tolerated, with a low complication rate, reinforcing its value in providing patients peace of mind regarding potential malignant transformation—especially in cases where tumors appear FDG-avid on PET scans, which may be a source of anxiety for patients despite benign pathology identified on FNA. While malignant transformation is well documented in the literature for pleomorphic adenomas,^22^ there is no solid evidence that this is a potential outcome for Warthin's tumors outside of a pre-operative misdiagnosis on FNA. Since the risk of malignant transformation in WTs is extremely low, often considered practically nonexistent, patients and physicians can make an informed, shared decision about whether surgery is appropriate, reserving it for cases involving cosmetic concerns, prior or recurrent infections, and local pain or discomfort.^23^

The novelty of our study lies in its delineation of the distribution of benign parotid gland tumors in Veterans and provides clinically actionable evidence of surgical complications, recurrence, FNA–final pathology concordance, and PET-avidity interpretation with WTs. This Veteran specific data informs surgical decision-making and addresses a gap in the literature. Future studies should include multi-institutional Veteran specific data sets and include civilian cohorts to enable direct comparisons and external validation.

The study's limitations include its single-institution data source and a predominantly male population, limiting generalizability. Additionally, the retrospective nature of this study, we introduced potential selection and information bias due to record incompleteness. To mitigate bias, we applied consistent inclusion criteria, excluded non-diagnostic FNAs, and relied on standardized documentation from otolaryngology clinic notes and EMR data extraction. Post-operative follow-up was short and likely underestimating potential follow-up and limiting detection of late outcomes. Despite these limitations, given the unique risk factors and high tobacco use in the Veteran population, understanding variations in parotid pathology remains clinically relevant, making this paper important.

## Conclusion

In this Veteran and predominantly male cohort, Warthin's tumor was the most prevalent parotid neoplasm and strongly associated with heavy tobacco use. The high concordance between FNA and surgical pathology underscores the expertise of cytopathologists and pathologists in this patient population. This pre-operative and surgical pathology agreement may provide surgeons with confidence when counseling patient's pre-operatively and discussing the surgical management of this entity. Future studies should include multi-center veteran institutions to better capture population trends and enable direct comparison of risk factors between Veterans and civilians.

## Supplemental Material

sj-docx-1-sci-10.1177_00368504251399563 - Supplemental material for Tobacco exposure linked to Warthin's tumor being the most common benign parotid neoplasm in Veterans: A retrospective cohort study Supplemental material, sj-docx-1-sci-10.1177_00368504251399563 for Tobacco exposure linked to Warthin's tumor being the most common benign parotid neoplasm in Veterans: A retrospective cohort study by Louis Abarca, Linda K. Green, Ray Y. Wang, Vlad C. Sandulache and David J. Hernandez in Science Progress
