# Supplementary material for: Tobacco exposure linked to Warthin's tumor being the most common benign parotid neoplasm in Veterans: A retrospective cohort study
Source: Sci Prog. 2025 Nov 18;108(4):00368504251399563. doi: 10.1177/00368504251399563 (PMC12627354; doi:10.1177/00368504251399563)
Supplement: sj-docx-1-sci-10.1177_00368504251399563 - Supplemental material for Tobacco exposure linked to Warthin's tumor being the most common benign parotid neoplasm in Veterans: A retrospective cohort study [file sj-docx-1-sci-10.1177_00368504251399563.docx]

| **Table S1:** Smoking History of Top 3 Diagnoses in FNA only and Surgical Patients | | | | |
| --- | --- | --- | --- | --- |
| **Histology Type** | **FNA Only n/N (%)** | **p value (FNA-only)** | **Surgical n/N (%)** | **p value (Surgical)** |
| Warthin Tumor | 58/68 (85.3) | < 0.05 | 39/46 (84.8) | < 0.05 |
| Pleomorphic Adenoma | 0 | - | 12/24 (50.0) | - |
| Lymphocytes | 14/30 (46.7) | - | 0 | - |
| Cyst | 12/25 (48.0) | - | 5/8 (62.5) | - |

Note: p-values, comparing Warthin tumor vs all other most common histologies within each cohort.
